# Insect herbivory facilitates the establishment of an invasive plant pathogen

**DOI:** 10.1038/s43705-021-00004-4

**Published:** 2021-03-22

**Authors:** Martin M. Gossner, Ludwig Beenken, Kirstin Arend, Dominik Begerow, Derek Peršoh

**Affiliations:** 1 Forest Entomology, Swiss Federal Research Institute WSL, Birmensdorf, Switzerland; 2 Terrestrial Ecology Research Group, Department of Ecology and Ecosystem Management, Center for Food and Life Sciences Weihenstephan, Technische Universität München, Freising-Weihenstephan, Germany; 3 ETH Zurich, Department of Environmental Systems Science, Institute of Terrestrial Ecosystems, Zurich, Switzerland; 4 Forest Protection, Swiss Federal Research Institute WSL, Birmensdorf, Switzerland; 5 Ruhr-Universität Bochum, Faculty of Biology and Biotechnology, AG Geobotany, Bochum, Germany

**Keywords:** Forest ecology, Forest ecology

## Abstract

Plants can be severely affected by insect herbivores and phytopathogenic fungi, but interactions between these plant antagonists are poorly understood. We analysed the impact of feeding damage by the abundant herbivore *Orchestes fagi* on infection rates of beech (*Fagus sylvatica*) leaves with *Petrakia liobae*, an invasive plant pathogenic fungus. The fungus was not detected in hibernating beetles, indicating that *O. fagi* does not serve as vector for *P. liobae*, at least not between growing seasons. Abundance of the fungus in beech leaves increased with feeding damage of the beetle and this relationship was stronger for sun-exposed than for shaded leaves. A laboratory experiment revealed sun-exposed leaves to have thicker cell walls and to be more resistant to pathogen infection than shaded leaves. Mechanical damage significantly increased frequency and size of necroses in the sun, but not in shade leaves. Our findings indicate that feeding damage of adult beetles provides entry ports for fungal colonization by removal of physical barriers and thus promotes infection success by pathogenic fungi. Feeding activity by larvae probably provides additional nutrient sources or eases access to substrates for the necrotrophic fungus. Our study exemplifies that invasive pathogens may benefit from herbivore activity, which may challenge forest health in light of climate change.

## Introduction

Insect herbivores and phytopathogenic fungi can both have severe impacts on individual plants, but also on entire plant populations.^1–4^ The magnitude of these impacts is often modified by insect-fungus interactions,^5–10^ which act directly or indirectly.^11^ Indirect interactions are mostly plant-mediated, i.e. attacks stimulate the defence mechanism in plants, which results in induced resistance to subsequent attacks.^12–14^ Induced susceptibility appears less common,^15^ in particular for herbivory-induced susceptibility to fungal pathogens.^11^ Herbivore activity may, however, also directly affect the susceptibility to fungal colonisation locally.^16^ This includes the facilitation of fungal infection by physical damage of host tissue^8^ and by enhancing fungal growth due to increased availability of nutrients supplemented or made accessible by insects,^17,18^ for instance by honeydew accumulation on leaves^19^ or frass giving nutritional substrates to fungi.^18^

Reproduction and dispersal of many phytopathogenic and endophytic fungi rely on insect vectors.^15,20–22^ For tree species, this is best known from Dutch elm disease, which was caused by a fungus (*Ophiostoma ulmi* (Buisman) Nannf.) vectored by bark beetles.^23^ Insect vectors have also been suggested for other pathogens such as the agent of Ash dieback (*Hymenoscyphus fraxineus* (T. Kowalski) Baral, Queloz & Hosoya^24^) and are well known in transmitting the thousand cankers disease of walnut^25^ and blue stain fungi.^26^ The establishment of invasive pathogens on novel hosts in their new environment may be facilitated by the occurrence of species congeneric with their native hosts, to which pathogens are likely to be preadapted.^27–29^ Understanding the mechanisms underlying the spread and infection pathways of introduced pathogens in their new environment is strongly required, in particular for invasive fungi potentially causing devastating tree diseases.

**Fig. 1:**
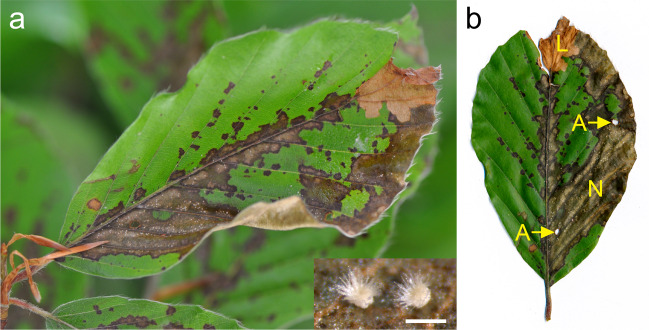
Appearance of studied plant-insect-fungus interaction. Shade leaf of *Fagus sylvatica* under natural conditions (**a**) and as a detached leaf (**b**). The leaf shows feeding traces of larvae (L) and adults (A) of *Orchestes fagi*, as well as necroses (N), caused and mycopappus-like propagules (white spots; enlarged in the insert of **a**, bar = 0.2 mm) formed by *Petrakia liobae*.

Here, we focus on the pathogenic fungus *Petrakia liobae* Beenken, Andr. Gross & Queloz, which was recently described as a species new to science, occurring on European beech, *Fagus sylvatica* L.^30^ The species has only recently appeared in Central Europe and was first mistaken as being the Japanese species, *Pseudodidymella fagi* C.Z. Wei, Y. Harada & Katum.^30,31^ *P. liobae* causes blackish necroses on living beech leaves, on which striking white, so-called mycopappus-like propagules are formed for asexual dissemination (Fig. 1). Since these conspicuous symptoms were never reported before 2008, the pathogen is certainly not native to Central Europe. Although the geographical origin of *P. liobae* remains unclear, the species can be considered invasive in Central Europe.^30^

Since its first detection in Switzerland in 2008, necroses caused by *P. liobae* have been increasingly observed in different countries of Central Europe and the fungus currently appears to be spreading northwards.^30^ While detailed information on its invasion history is lacking, this scenario is supported by the data from a metabarcoding approach.^32^ In this study, Guerreiro et al.^32^ detected DNA of ‘OTU 3’, which is nowadays identifiable as *P. liobae* and accounted for 14% of the endophytic fungi in beech leaves of the Swabian Alb (southern Germany) in autumn 2014. At the same time, it accounted for 2% of the endophytes in central (i.e. Hainich-Dün) and for <1% in northern Germany (i.e. Schorfheide-Chorin).

*P. liobae* might be preadapted to *Fagus*, but preadaptations of the new host to the invasive pathogen is unlikely because pathogens related to *P. liobae* are not known from European beech so far. Evolutionary adaptation of European beech to *P. liobae* is also unlikely due to the recent encounter.^31,33^ We, therefore, consider specific co-evolutionary mechanisms to play a minor role in the defence of European beech populations against *P. liobae*. Predominantly non-specific defence mechanisms, such as physical protection, are expected to control infection rates. Consequently, *P. liobae* might be less successful in invading sun leaves of European beech, which possess thicker outer cell walls,^34,35^ than the physically less protected shade leaves. Feeding herbivorous insects may, however, decrease the difference in pathogen infection between sun and shade leaves caused by physical protection.

One of the most common and abundant insect herbivores on European beech leaves is the weevil *Orchestes fagi* L. It has also been observed to co-occur with *P. liobae* (Fig. 1). It mainly occurs in the canopy and its population density decreases from south to north in Germany.^36^ After overwintering in bark crevices and in the soil and leaf litter, maturation feeding by adults causes characteristic round holes (Fig. 1) in the leaves in spring, mainly on shaded leaves,^34^ where also the eggs are laid near the midveins. Larval feeding causes characteristic gradual widening serpentine tunnel mines towards the leaf edge where a large irregular blotch mine is built in which the larvae pupates. Emerging adults of the new generation prefer sun-exposed leaves for feeding,^34,37^ leading to an overall higher herbivory rate in the upper canopy later in the year.^36^ The species shows pronounced population fluctuations among years and can cause incremental losses and substantial losses in beech mast during outbreaks.^38,39^

**Fig. 2:**
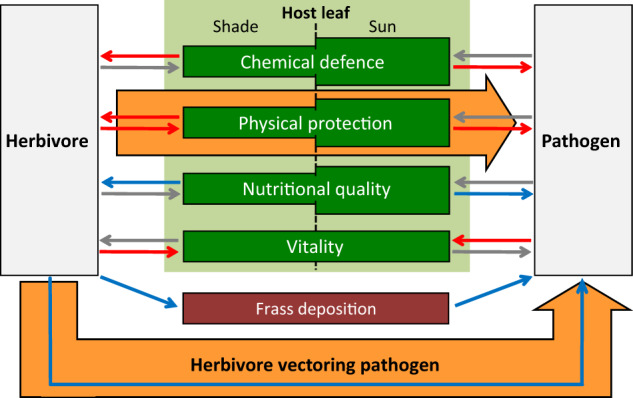
Potential interactions between *Orchestes fagi* and *Petrakia liobae*. Schematic drawing indicating direct and indirect, i.e. host plant-mediated, effects of the herbivore on the pathogen. Involved plant properties are grouped as biochemical responses (chemical defence) and physical protection for defence-related traits. Nutritional quality and vitality sum leaf traits of major relevance for herbivore and pathogen selection. Red arrows indicate negative effects, blue arrows positive effects and grey arrows effects of unknown or ambivalent type (e.g. herbivores might induce chemical defence or reduce overall defence capacities). Heights of the green boxes indicate relative markedness of traits in shade and sun leaves. Hypotheses tested in this study are depicted by orange block arrows.

We combined a field survey of feeding traces with metabarcoding of endophyllous mycobiomes to test the hypothesis that infection rates of beech leaves with *P. liobae* increase with feeding damage caused by *O. fagi* (Fig. 2). Fungi associated with hibernating *O. fagi* beetles were assessed in early spring, before bud burst, to test if the beetles are potential vectors of the fungus. Although induced susceptibility to subsequent attacks is less common than induced resistance (see above), we assume that insect herbivore feeding is likely to directly affect fungal colonisation due to the creation of entry ports (Fig. 2, ‘physical defence’). We analysed mycobiome composition and relative abundance of *P. liobae* in leaves from the uppermost south-faced sun-exposed canopy and shaded canopy (henceforth referred to as sun and shade leaves) of each tree separately, because we assumed that the effect of herbivory on endophytic fungi is more pronounced in sun leaves. Sun leaves are supposed to possess higher physical and chemical defence capabilities than shade leaves.^40–42^ Herbivore feeding predominantly affects the physical defence (Fig. 2), but induced chemical plant defence may also be relevant. However, these two mechanisms are difficult to separate in field studies. In order to study how plant physical defence affects fungal infection, we complemented our field studies by a laboratory experiment comparing infection success of the fungus in intact and artificially perforated detached sun and shade leaves under controlled conditions. By doing this we aimed to exclude animal-derived elicitors as well as systemic responses of beech.

## Materials and methods

### Study sites

Sampling of beetles and beech leaves (*Fagus sylvatica* L.) for mycobiome analyses was conducted on a 100 m × 100 m beech forest plot in the Swabian Alb, near Münsingen (48°22′57″N; 9°22′57″E), Germany. The plot is part of the Biodiversity Exploratories Project^43^ and was selected because *P. liobae* was reported earlier (as OTU 3) from that plot.^32^ The elevation is 766 m a.s.l., the average temperature in 2 m height between 2010 and 2017 was 7.6 °C (data from project climate station) and average annual precipitation (based on RADOLAN RW product, see www.dwd.de/RADOLAN; DWD Climate Data Center (CDC)) between 2006 and 2017 was 963.9 mm. The forest originates from a former woodland pasture and is characterised by a mix of beech trees of different ages, including some old, structurally rich beech trees with large crowns.

For the final laboratory experiment (conducted in the Plant Protection lab at WSL, Birmensdorf, Switzerland), we used a strain of *P. liobae* that was isolated previously and deposited in the culture collection of the Westerdijk Fungal Biodiversity Institute (CBS), Utrecht, the Netherlands (CBS 145956).^30^ Beech leaves were collected in a beech forest mixed with old oak (*Quercus robur* L.) trees from former coppice with standard forestry and fir (*Abies alba* Mill.) trees after checking the forest for *P. liobae* infections. The forest is located near Utikon Waldegg, Switzerland (47°21′57″N; 8°28′08″E) at 630 m a.s.l., the average temperature in 2 m height between 2006 and 2016 was 9.8 °C and average annual precipitation 1072.7 mm (data from MeteoSchweiz, Swissmetnet, interpolated by Meteotest).

### Mycobiome of *Orchestes fagi*

Evidence for *O. fagi* vectoring *P. liobae* was searched by screening the fungi associated with hibernating individuals of *O. fagi* for the presence of *P. liobae*. Thirty beetles were collected from the bark of seven different trees along a 100 m transect across the plot and two from litter around these trees on 20 March 2015. The beetles were stored at approximately 4 °C in plastic vessels equipped with a crumpled paper towel. In the laboratory at Bochum, Germany, half of the beetles were transferred to 0.2 ml tubes with 100 µl double-distilled sterile water (ddH_2_O). Subsequently, they were transferred twice to 0.5 ml ddH_2_O in 1.5 ml tubes and washed by vortexing (5 s at 3200 rpm) another two times to remove loosely attached fungal spores. From ten of the beetles and the corresponding washing waters, which were concentrated in a centrifugal evaporator (at 50 °C for 50 min) beforehand, DNA was extracted using the Charge Switch® gDNA Plant Kit (Invitrogen) as detailed by Werner et al.^44^ The other six beetles and 50 µl of the respective washing waters were plated on YM media [(1.0% (w/v) Glucose, 0.3% (w/v) Yeast extract, 0.5% (w/v) Peptone, of 0.3% (w/v) Malt extract and 2.0% (w/v) Agar)] supplemented with 100 mg/ml Chloramphenicol. The plates were observed daily and emerging fungal mycelia were transferred to new plates. DNA was extracted from pure cultures (fragments about 12 mm^3^) using the Charge Switch® gDNA Plant Kit (Invitrogen) as recommended by the manufacturer, but with all volumes reduced to 10%.

### Sampling of beech leaves for assessing feeding damage by *Orchestes fagi* and mycobiome analyses

Beech leaves were sampled from a total of 43 trees distributed across the entire forest plot (see above) between 28 June and 4 July 2015. We focused on trees of the upper canopy layer only (dbh: mean 42.7 ± 3.8 SE) and randomly selected 43 trees from a total of 200 based on previous surveys. Following a paired sampling design, two branches were collected from both, the sun-exposed (uppermost south-faced) canopy and shaded canopy of each tree. The branches were sampled by shooting a rope over a small branch using a crossbow. By pulling on both ends of the rope the branch was broken off (for details, see ref. ^36^) For verification that leaves sampled in the two vertical strata of the canopy indeed represented sun and shade leaves, according to the definition given in botanical textbooks,^35^ we used eye (leaf size) and feel (leaf toughness) in the observational study and measured cell wall thicknesses of all leaves used in the lab experiment, as detailed below.

For assessing herbivory caused by adult *O. fagi* on sun and shade leaves, 50 leaves of each of the two branches were sampled per tree. We found the typical circular chewing damage caused by adult beetles of *O. fagi* in spring and assessed the herbivory rate of this damage type as a proportion of leaf area loss by naked-eye using a series of templates representing beech leaves with round holes reflecting a different percentage of leaf area loss.

The endophytic mycobiome was assessed in 240 leaves (Supplementary Table S1). Therefore, five leaves of each of the following categories were sampled from the two branches of each sun-exposed and shaded canopy, when available: (1) 36 sun and 40 shade leaves, respectively, without feeding damage of *O. fagi* or any other herbivore; (2) 42 sun and shade leaves each with exclusively damage of adult *O. fagi* (small round holes); (3) 38 sun and 42 shade leaves with damage of adult *O. fagi* plus mines caused by larval frass of *O. fagi*, but without signs of other herbivores.

Subsequently, five sun and shade leaves, respectively, from each tree and each feeding damage category were pooled and surface sterilised in the field using a series of H_2_O, 70% EtOH and 1.2% NaOCl as detailed by Peršoh et al.^45^ Twenty discs of 7 mm in diameter were cut from the five leaves with a cork borer and transferred for immediate desiccation to a 15 ml tube, half-filled with silica gel. In the laboratory at Bochum, Germany, DNA was extracted from dried leaf disks using the Charge Switch® gDNA Plant Kit (Invitrogen) as detailed above for cultures. Cell disruption and lysis were achieved by incubation of the discs in 2 ml tubes with 100 μl kit lysis buffer and glass beads (0.06 g of 0.25–0.5 mm diam., 0.03 g of 0.1–0.25 mm diam. and 5 beads of 1.25–1.55 mm diam.) at 5 m/s for 60 s in a FastPrep device (FastPrep®-24, MP Biomedicals).

### Library preparation and sequencing

Sanger sequencing of the ITS barcoding region of fungal strains followed Greiner et al.^46^ Amplicon libraries were prepared from DNA extracts from beetle and leaf samples as detailed by Guerreiro et al.^32^ Briefly, the fungal ITS region was amplified in two consecutive PCR reactions, with TAG and index sequences introduced in the first (PCR1) and second PCR (PCR2), respectively. The primer combination ITS1F^47^ and ITS4^48^ was used with the GoTaq® G2 Hot Start Colorless Master Mix (Promega) for selective amplification of the fungal ITS rRNA gene region in PCR1 (3 min at 95 °C, 33 × (94 °C for 27 s, 57 °C for 60 s and 72 °C for 90 s), 7 min at 72 °C). Sequences of the sequencing primers, Illumina indices and adapters were added in PCR2 (6 × (94 °C for 27 s, 53 °C for 60 s and 72 °C for 90 s)) to 5 µl of purified (Rapid PCR Cleanup Enzyme Set kit, New England Biolabs) PCR1 product. After equimolar pooling of purified PCR products, DNA concentration was assessed with a Qubit® 2.0 fluorometer (Life Technologies, Carlsbad, USA) and the library was sequenced using an Illumina MiSeq sequencer (Illumina Inc., San Diego, USA) with 2 × 250 bp paired-end sequencing (MiSeq Reagent Kit v3 Chemistry, Illumina Inc.). The sequence reads were processed as detailed by Röhl et al.^49^ Briefly, the sequence reads were quality filtered and demultiplexed (i.e. assigned to samples) using the Qiime pipeline.^50^ Only the forward reads (covering the ITS1 region of the rRNA gene) were further processed. After trimming, the sequences comprised 174 bp of the rRNA gene, including 14 bp of the SSU and 160 bp upstream (i.e. ITS1 plus partial 5.8 s region). These sequences were clustered to operational taxonomic units (OTUs) by applying a 97% similarity threshold and using CD-HIT OTU for Illumina (v.0.0.1).^51,52^ Based on representative sequences, OTUs were provisionally assigned to taxa using the UNITE database^53^ as reference. OTUs corresponding to *P. liobae* were identified by 100% sequence similarity with the recently published reference sequences.^31^

### Quantitative PCR

The ratio of fungus to host DNA was assessed by qPCR comparatively among the 6 leaf categories (leaf type × feeding damage). Species-specific primers were designed which amplify a 67 bp fragment of the ITS1 region of *P. liobae* (Pfagi_f01: 5′-ATCATTACCGTGGGGATTCG-3′, Pfagi_r01: 5′-GAGGAAACGAGGGTACTCATG-3′) and 107 bp fragment of the ITS2 region of *F*. *sylvatica* (Fa_f02: 5′-TTTGGTGGCGGAAGTTGG-3′, Fa_r02: 5′-GACCGAGGTCTAATCAACCAC-3′), respectively. Of the DNA extracts used for diversity assessment (i.e. amplicon metabarcoding) as detailed above, 15 extracts from leaves of each of the 6 categories were selected for the qPCR approach. Fungus and host DNA were quantified in independent reactions, but using aliquots of the same DNA extract. Each qPCR reaction included 4 µl of the Luna® Universal qPCR Master Mix, 1 µl of primers (i.e. 0.5 µl of each of the two primers amplifying *P. liobae* or *F*. *sylvatica*, respectively) and 3 µl of DNA extract (1:4 diluted). The reaction was run on a LightCycler® 480 (Roche) and included an initial denaturation (95 °C for 60 s) followed by 40 amplification cycles (95 °C for 15 s, 60 °C for 30 s + plate read) and melting curve assessment.

### Laboratory experiment

The strain of *P. liobae* was plated and cultivated on 1.5 % (w/v) malt extract agar (MEA) amended with 100 mg/l streptomycin and incubated at 20 °C in the dark.^30^ Emerging mycelium was multiplied to obtain enough active fungal mycelium for the experiments. We verified the identity of the fungal mycelium to be *P. liobae* by sequencing the ITS region as described in Beenken et al.^30^ In a pilot experiment, we verified that the obtained cultivated mycelium can infect beech leaves and induce necroses bearing the typical mycopappus-like propagules of *P. liobae.*^31^

For experimental infection, we sampled the sun-exposed and shaded canopy of 10 mature beech trees, as described above. Details on the experimental procedures are given in the supplementary information. Briefly, of each tree and leaf type (sun vs. shade leaves) 15 visually healthy and undamaged leaves were chosen and cleaned carefully with sterile water to keep the outer cuticula and cell walls intact. The experiment comprised three treatments: one-third of the leaves (i.e., 5 leaves per tree and leaf type) was perforated to simulate physical herbivore feeding damage, a commonly used method in plant pathology,^54–56^ and inoculated with *P. liobae*. One third was inoculated but not perforated and one third was perforated but not inoculated (control). The leaves were incubated separately in sterile, transparent plastic boxes at 100% relative humidity at 20 °C under permanent light. Leaf condition was observed twice per week and necrotic areas were quantified using the open-source programme ImageJ,^57^ based on digital images taken after 11 days of incubation. As measures of *P. liobae* infection, we used three different parameters: (1) proportion of leaves infected, (2) time until necrosis appears, and (3) size of the necrosis after 11 days.

The thickness of the upper outer epidermal cell wall (including the cuticula) of one sun and one shade leaf from each of the 10 trees were measured microscopically, conducting four measurements per leaf from 5 to 10-µm-thick sections of leaf pieces cut for the perforation treatments.

### Data analyses

All analyses were conducted in R version 3.3.1^58^ as detailed in the supplementary information. Briefly, we used linear mixed-effects models to test for differences in herbivory in the field study, in the thickness of the outer cell walls and in signs of *P. liobae* infection in the laboratory experiment. We calculated a PERMANOVA based on Bray–Curtis dissimilarity to compare endophyte mycobiome between leaf type × feeding damage category. Subsequently, we used the pairwise .adonis function to assess the contribution of particular fungal OTUs to the separation of combined categories by SIMPER-analyses. We indicated the fungus:host ratio from the qPCR data as ΔCp, i.e. by subtracting Cp-values of the fungus by those of beech. To test whether leaf type × feeding damage category affected the Cp-value for beech and to test for differences in ΔCp we used linear mixed-effects models and tested for differences among categories using Tukey contrasts. *R*^2^ values were calculated based on the extension^59,60^ as implemented in the package MuMIn.^61^

## Results

Feeding damage did not significantly differ between shade and sun leaves, neither by adult beetles of *O*. *fagi*, nor by their larvae (Supplementary Fig. S1 and Supplementary Table S2). The fungus *P. liobae* was neither present among the fungal strains isolated from hibernating beetles, nor among the 240,001 reads obtained from DNA extracts from the beetles (Supplementary Tables S1, S3 and S4). Amplification of fungal DNA was not successful for DNA extracts from the washing waters.

### Mycobiomes

A total of 219 fungal OTUs, represented by 4,714,323 sequence reads, were found in the 179 leaf samples covered by more than 5000 reads, each (Supplementary Tables S1, S3 and S4). The mycobiomes differed significantly among feeding damage categories (*F*_2,176_ = 3.818, *R*^2^ = 0.042, *p* < 0.001), but not between leaf types (*F*_1,177_ = 1.049, *R*^2^ = 0.006, *p* = 0.276) (PERMANOVA, Supplementary Table S5). The interaction between both factors was not significant (*F*_2,176_ = 1.123, *R*^2^ = 0.012, *p* = 0.249). Pairwise comparisons revealed that damage caused by larvae was associated with a change of the mycobiomes in the shade and sun leaves (adjusted *p* < 0.05), while adult damage was only associated (marginally not significant; adjusted *p* = 0.09) with mycobiomes change in sun leaves (pairwise Adonis, Table 1, Supplementary Table S6).

**Table 1:** Contribution of *P. liobae* to pairwise differences between leaves with different feeding damage according to mycobiome compositions.

Pairs of feeding categories	*F*-value	*R*^2^	*p*-value	Adjusted *p*-value	Contribution by *P. liobae* [%]
Shade					
No vs adult	1.41	0.023	0.127	1	14
No vs adult + larvae	3.61	0.053	0.001	0.015	18
Adult vs adult + larvae	1.96	0.027	0.025	0.375	20
Sun					
No vs adult	2.27	0.047	0.006	0.09	14
No vs adult + larvae	4.73	0.088	0.001	0.015	21
Adult vs adult + larvae	1.20	0.020	0.275	1	26

The results of the pairwise permutational multivariate analysis of variance (adonis) are given for pairwise comparisons of feeding categories of sun and shade leaves. The adjusted *p*-value represents the *p*-value after Bonferroni correction. The contribution of *P. liobae* to the total dissimilarity between mycobiomes according to SIMPER analysis (Similarity per cent) is given in the final column.

The difference between mycobiomes in leaves of different categories (feeding damage × leaf type) was mostly explained by a fungal OTU assigned as *P. liobae* (Table 1 and Supplementary Table S7). It contributed to the overall dissimilarity between 14 and 26%. *P. liobae* was least abundant in the mycobiomes of undamaged sun leaves (relative abundance 1%) and most abundant in sun leaves with feeding damage by adults and larvae of *O. fagi* (38%). In sun leaves only damaged by adult beetles, *P. liobae* was of intermediate abundance (26%). An increase in abundance of *P. liobae* with feeding damage was also observed in the mycobiomes of shade leaves, but this was less pronounced (undamaged: 11%, adult damaged: 20%, adult and larvae damaged: 31%).

### Pathogen–host ratio

**Fig. 3:**
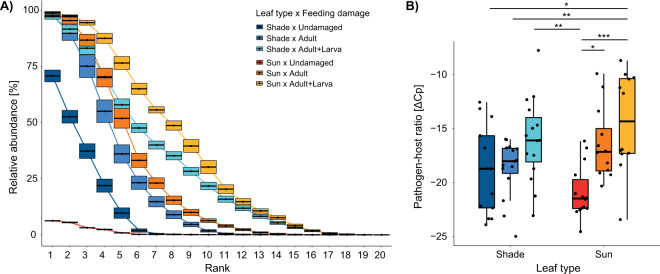
Relative abundance of *Petrakia liobae* in beech leaves. **a** Proportion of *P. liobae* in the total endophytic mycobiome. Means and 95% confidence intervals based on 100 re-samplings of 20 leaves each are shown. Samples were rank-ordered after each resampling and values of each rank were averaged across all re-samplings. **b** Pathogen–host ratio (median, box: 75%/25% quantiles, whisker: min/max values, dots: single values). The ratio of molecular markers is indicated by the difference of the Cp-values ascertained by qPCR. Leaf treatment: sun (reddish) and shade (blueish) leaves without (dark colours) feeding traces of *Orchestes* fagi, with feeding traces by adult beetles (intermediate brightness) and by traces of adult and larval beetles (bright colours). Significant pairwise differences according to post-hoc comparisons following the linear mixed-effects model (Supplementary Tables S8 and S9) are indicated by asterisks: *p < 0.05, **p < 0.01, ***p < 0.001.

The relative proportion of *P. liobae* in the mycobiomes differed significantly between the different leaf categories, with the lowest proportion in undamaged sun leaves and highest proportion in sun leaves that were damaged by both, *O. fagi* adults and larvae (Fig. 3a). By quantifying *P. liobae* relative to the leaf tissue (fungus:plant ratio) using qPCR we found consistent results (Fig. 3b). While the content of beech-DNA did not differ among categories (Supplementary Fig. S2 and Supplementary Table S8), the fungus was most abundant in leaves damaged by adults and larvae of *O. fagi*, followed by leaves damaged only by *O. fagi* adults and undamaged leaves (Fig. 3). This trend was visible in both, shade and sun leaves, but more pronounced (and only significant) in the sun leaves (Fig. 3 and Supplementary Tables S9, 10). We observed a marginal *R*^2^ (including only fixed effects) of 0.261 and a conditional *R*^2^ (including fixed and random effects) of 0.329. Thus, around 33% of the variation is explained by our fixed and random effects, which show that the relationship is quite strong.

### Incubation experiment

**Fig. 4:**
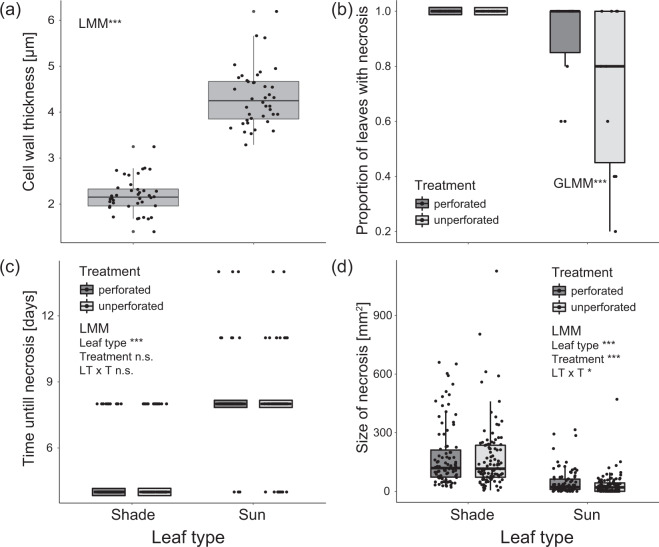
Incubation experiment. Properties of the incubated leaves from shade (Shade) and sun-exposed (Sun) canopy and results of the incubation. **a** Thickness of outer cell wall of upper epidermis cells in the shade and sun leaves (4 measurements per one sun and per one shade leaves from each of 10 trees). **b** Proportion of unperforated and perforated leaves from the shaded and sun-exposed canopy on which necrosis was observed (5 leaves per treatment and leaf type on each of 10 trees). **c** Time (number of days, assessed two times a week) until necrosis was observed on perforated and unperforated shade and sun leaves. Each leaf comprised two infection trials. Only trials in which necrosis were observed (*N* = 370 of 400 trials) are considered. **d** Infection extent (size of necrosis) after 11 days as a function of leaf type and treatment (*N* = 388 of 400 trials). Median, 25%/75% percentiles (boxes) and min-max values (whisker) are shown in addition to single data points. Significant differences according to post-hoc comparisons following linear mixed-effects models (LMM, Supplementary Tables S11, S13 and S14) or generalised linear mixed-effects models (GLMM, Supplementary Table S12) are indicated by asterisks: *p < 0.05, **p < 0.01, ***p < 0.001, n.s. not significant. For illustrative reasons, small boxes were drawn around the median in **b** (shade) and **c**.

Sun and shade leaves used for the experimental incubations differed significantly (*F*_1,69_ = 550.3, *p* < 0.0001) in the thickness of the outer cell wall of the epidermis cells of the upper leaf surfaces (Fig. 4a and Supplementary Table S11). Independent of leaf type (sun vs. shade leaves), no necrotic spots were observed on control leaves (punched, but not exposed to *P. liobae*) after 30 days of incubation. Necroses developed on all shade leaves in the same time period, when incubated with pure cultures of *P. liobae* (Fig. 4b). Sun leaves became less often symptomatic than shade leaves (Fig. 4b). Shade leaves developed necrosis in all leaves investigated, whether or not they were perforated prior to inoculation. In contrast, sun leaves developed necroses more often (*z*_1,194_ = 4.927, *p* < 0.0001) when perforated, as compared to non-perforated leaves (Fig. 4b and Supplementary Table S12). The time passed until necrosis was observed (i.e. incubation time) was not affected by perforation, neither in shade, nor in sun leaves (Treatment: *F*_1,317_ = 0.224, *p* = 0.636; Interaction treatment × leave type: *F*_1,317_ = 1.426, *p* = 0.223), but necrosis required a significantly longer (*F*_1,317_ = 495.367, *p* < 0.0001) period of time to develop on sun than on shade leaves (Fig. 4c and Supplementary Table S13). The size of the necroses was also significantly bigger (*F*_1,335_ = 395.166, *p* < 0.0001) on shade than sun leaves (Fig. 4d and Supplementary Table S14). Additionally, the size of the necroses was bigger in perforated than unperforated leaves, but only in sun leaves (treatment: *F*_1,335_ = 11.526, *p* < 0.001; Interaction treatment × leave type: *F*_1,335_ = 6.474, *p* = 0.011).

## Discussion

The invasive *Petrakia liobae* occurred inside a considerable proportion (88%) of the leaves of European beech at the study site (Supplementary Table S3), while disease symptoms were not observed in the field. Like the most common leaf pathogen of European beech, *Apiognomonia errabunda (Roberge ex Desm.) Höhn*., *P. liobae* appears to grow mostly asymptomatically inside the leaves, as an endophytic fungus.^62^ However, necroses of both pathogens have been increasingly observed, emphasising the importance of these pathogens on beech.^30^

Our study showed a positive correlation between herbivore damage and infection success by the invasive pathogen *P. liobae*. Co-evolutionary mechanisms have not developed in interactions with recently encountered invasive pathogens,^33^ which renders specific plant-mediated interactions improbable to underlie our findings. They may thus be explained by the following mechanisms, i.e. (1) herbivores function as vectors for the pathogen, (2) herbivore and pathogen occurrences on leaves are driven by the same factors, (3) herbivore feeding promotes pathogen infection, or vice versa. First, the most important herbivore on beech, i.e. *Orchestes fagi*, most likely does not serve as a vector, as overwintering beetles did not carry *P. liobae* shortly before bud burst. We cannot exclude that *O. fagi* could transmit the fungus among leaves through feeding activity and frass deposition by adults (might be intercepted by leaves) and less likely larvae (larval frass and faeces accumulate in the mines until beetle emergence) and thus amplify it in the host population. However, we would expect that it should then be also detectable in overwintering adults making it less likely that the beetle serves as a long-distance vector. Second, preferences of herbivore and pathogen for the same leaves are unlikely to explain their interaction, because of significantly different responses of the pathogen to feeding damage in sun and shade leaves (Fig. 3) in contrast to negligible differences in the extent of feeding damage (Supplementary Fig. S1). Third, the fungus was less abundant in leaves damaged by only adult feeding than in leaves with adult and larval feeding traces (Fig. 3). A potential positive impact of the fungus would therefore appear either beneficial for both growth stages of the beetle, or the adults deposit their eggs preferentially in infected leaves to take advantage of a potential beneficial effect of the fungus for larval development. Such symbiotic interactions may not be excluded, but are unlikely to have already developed in interactions with recently encountered invasive pathogens.^33^ It, therefore, appears more likely that herbivore feeding impacts pathogen infection, and not vice versa. This applies particularly with regard to rather simple and therefore more parsimoniously conceivable relations. The two mechanisms most likely underlying the observed facilitation of infections of the invasive pathogen by the native insect herbivore are (1) induced susceptibility and (2) impairment of the physical defence of the host.

Previous studies were inconsistent regarding the effects of feeding by herbivores on susceptibility and resistance of the host plant to fungal infection, with 23% of the studies showing a positive association, 31% a negative association and 46% had no effect or a variable effect.^63^ In our field study, leaves with feeding traces by *O. fagi* were more often infected and infected to a higher degree by *P. liobae* than undamaged leaves (Fig. 3), indicating that feeding activity of *O. fagi* did not induce a higher resistance to *P. liobae*. Infection success of the fungus further increased with the extent (diversity) of feeding damage, i.e. leaves with feeding traces by larvae and adults were infected to a higher degree by *P. liobae* than leaves with traces by adult feeding only. These results would be accordable with induced susceptibility of the host plant to *P. liobae* by herbivore feeding. The higher herbivore-induced responses of *P. liobae* in sun compared to shade leaves might suggest that the effect of herbivore feeding on colonisation by *P. liobae* is stronger in sun than in shade leaves (Fig. 3). Focusing on physical protection as an alternative and/or additive mechanism, the experimental data provide a more parsimonious explanation for our findings.

In the laboratory experiment, the fungus formed necrotic spots on all shade leaves (Fig. 4b), indicating that neither physical nor physiological host defence prevent a fungal infection of shade leaves under the experimental conditions. Although the experiment was conducted under optimal conditions for fungal growth (high moisture, additional nutritional source) the infection success was much lower in sun leaves and lowest in undamaged sun leaves (Fig. 4b, c). Mechanical leaf perforation, which was applied manually to detached leaves to minimise physiological plant responses (see above), increased infection success much more in sun leaves (Fig. 4b, c). Furthermore, the difference between infection rates of undamaged and damaged leaves was more pronounced in sun leaves in the field, as compared to shade leaves. All these results support the importance of physical protection against the fungal pathogen. The thicker epidermal cell walls in sun leaves (Fig. 4a) might lower infection success by the fungus in undamaged leaves. By breaking the mechanical barrier the herbivore provides entry ports for fungal infection. This is in agreement with the observation that *P. liobae* mainly affects beech leaves on low-hanging branches and young trees in the undergrowth of dense shady forests with high moisture in the field.^31,64^ However, feeding damage also provides access for the fungus to the interior of sun leaves. A direct interaction between herbivores and pathogens was already observed in other study systems.^8^ We assume that feeding damage by *O. fagi* adults, to physically better protected sun leaves in particular, can serve as an entry port for the *P. liobae* and thus indirectly promote its spreading. In the field, herbivore feeding in the sun leaves might be even more critical for opening the leaves to fungal infection as the microclimate is unfavourable for fungal growth in the sun-exposed canopy.

Feeding activity of leaf-mining larvae inside the leaves facilitated fungal infections to similar degrees in the sun and shade leaves when compared to the effect of feeding damage by adult beetles (externally chewing holes in leaves) only (Fig. 3). In contrast to feeding by adult beetles, the larval activity provides no additional entry port for the fungus into the leaf tissue, unless the mining tissue can be entered more easily by the fungus. It may provide additional nutrient sources for *P. liobae*, or ease access to nutrients. Deposition and accumulation of larval frass, which includes easily accessible nutrients, inside mines were shown to facilitate the growth of saprobic and pathogenic fungi inside infected plant tissues.^18^ *P. liobae* is a necrotrophic fungus,^31^ using devitalised but ultimately dead plant tissues as an energy source. Larval feeding also kills plant tissues, thus providing a probably suitable substrate for the fungus. Considering that *P. liobae* mostly occurs as an asymptomatic endophyte inside the leaves suggests that it is not generating notable amounts of the substrate (i.e. necrotic tissue) on its own. During its asymptomatic growth, the fungus may therefore benefit from tissues becoming necrotic due to the activity of other organisms. Such mechanisms have not been studied in detail, but maybe quite common considering the close evolutionary relationship between endophytic and necrotrophic lifestyles of fungi.^65^

Our results indicate that leaf toughness may play a major role in the defence of host plants against invasive leaf pathogens. We suggest that susceptibility to invasive pathogens is negatively correlated to physical protection and this should function as a working hypothesis for future studies. Our study further exemplifies that an invasive pathogen can profit substantially from the feeding activity of insect herbivores. While the multidisciplinary approach allowed us to infer plausible explanations for our findings, future studies should also assess chemical plant defence^66^ and focus on potential benefits for herbivores by endophytic fungi.^67,68^

The damage caused by herbivores^69–71^ and the occurrence of invasive pathogens^72,73^ are expected to increase in importance under climate change. Consequently, the interaction between native and invasive herbivores and pathogens might greatly challenge future forest health. Our study shows that interdisciplinary research is urgently needed to untangle the complex plant–pathogen–herbivore interactions in order to provide a scientific basis for developing management strategies to optimise forest health.

## Supplementary information

Supplementary Information

Supplementary Information

Table S1

Table S3

Table S4

## Data Availability

Sequence reads were deposited in the European Nucleotide Archive (http://www.ebi.ac.uk/ena) under accession PRJEB27000.
