# Supplementary material for: Insect herbivory facilitates the establishment of an invasive plant pathogen
Source: ISME Commun. 2021 Mar 22;1:6. doi: 10.1038/s43705-021-00004-4 (PMC9723786; doi:10.1038/s43705-021-00004-4)
Supplement: Supplementary file 1 — Supplementary Information [file 43705_2021_4_MOESM1_ESM.docx]

**Legends for supporting information**

**Figure S1**: Extent of feeding damage by adults *Orchestes fagi* on sun and shade leaves.

**Figure S2**: Quantity of beech-DNA across leaf categories.

**Table S1**: Details on the origin of the field samples analysed for mycobiome composition in the Illumina metabarcoding approach.

**Table S2**: Differences in herbivory by *Orchestes fagi* between sun and shade leaves.

**Table S3**: OTU table.

**Table S4**: OTU sequences and assignment of OTUs to taxa according to the UNITE database.

**Table S5**: Impact of leaf type (sun vs. shade leaves) and feeding damage category on compositional differences of endophytic mycobiomes.

**Table S6**: Pairwise differences in mycobiome composition between leaf categories.

**Table S7**: Contribution of OTUs to compositional differences of mycobiomes in leaves with different feeding damage.

**Table S8**: Differences in DNA content of the host between leaf categories.

**Table S9**: Differences in fungus:host ratio between leaf categories.

**Table S10**: Pairwise differences in fungus:host ratio between leaf categories.

**Table S11**: Differences in the thickness of cell walls between sun and shade leaves.

**Table S12**: Differences in the occurrence of necrosis between perforated and unperforated sun leaves.

**Table S13**: Differences in the time until necrosis was observed in the different leaf categories.

**Table S14**: Differences in the area affected by necrosis in the different leaf categories.
