# Supplementary material for: Insect herbivory facilitates the establishment of an invasive plant pathogen
Source: ISME Commun. 2021 Mar 22;1:6. doi: 10.1038/s43705-021-00004-4 (PMC9723786; doi:10.1038/s43705-021-00004-4)
Supplement: Supplementary file 2 — Supplementary Information [file 43705_2021_4_MOESM2_ESM.pdf]

Supplementary information for

Title: Insect herbivory facilitates the establishment of an invasive plant pathogen

Running title: Insect herbivory promotes invasive plant pathogen

Authors:

Martin M. Gossner[1,2,3*], Ludwig Beenken[4], Kirstin Arend[5], Dominik Begerow[5], Derek Peršoh[5*]

Author Affiliations:

[1] Forest Entomology, Swiss Federal Research Institute WSL, Zürcherstrasse 111, CH-8903 Birmensdorf, Switzerland

[2] Terrestrial Ecology Research Group, Department of Ecology and Ecosystem Management, Center for Food and Life Sciences Weihenstephan, Technische Universität München, Hans-Carl-von-Carlowitz-Platz 2, 85354 Freising-Weihenstephan, Germany

[3] ETH Zurich, Department of Environmental Systems Science, Institute of Terrestrial Ecosystems, 8092 Zurich, Switzerland

[4] Forest Protection, Swiss Federal Research Institute WSL, Zürcherstrasse 111, CH-8903 Birmensdorf, Switzerland

[5] Ruhr-Universität Bochum, Faculty of Biology and Biotechnology, AG Geobotany, Universitätsstraße 150, 44801 Bochum, Germany

* **Corresponding authors**:

Derek Peršoh (mycological part): E-mail: derek.persoh@rub.de, phone +49 234 32 27936

Martin M. Gossner (entomological part): E-mail: martin.gossner@wsl.ch, phone: +41 44 739 2588

The supplementary information includes detailed methods and results on the following topics.

Content

Mycobiome analyses ... 3

Table S5: Impact of leaf type (sun vs. shade leaves) and feeding damage category on compositional differences of endophytic mycobiomes. ... 4

Table S6: Pairwise differences in mycobiome composition between leaf categories. ... 4

Table S7: Contribution of OTUs to compositional differences of mycobiomes in leaves with different feeding damage ... 4

Quantitative PCR analyses ... 8

Figure S2: Quantity of beech-DNA across leaf categories ... 8

Table S8: Differences in DNA content of the host between leaf categories. ... 9

Table S9: Differences in fungus:host ratio between leaf categories. ... 9

Table S10: Pairwise differences in fungus:host ratio between leaf categories. ... 9

Differences in herbivory between sun and shade leaves ... 10

Figure S1: Probabilities (means, CI) of the extent of feeding damage by adults of *Orchestes fagi* (left) as well as probability of attack by adults, larvae and combined attack of adult/larvae of *Orchestes fagi* (right) on sun (N=2747) and shade (N=2146) leaves ... 10

Table S2: Differences in herbivory by *Orchestes fagi* between sun and shade leaves ... 10

Incubation Experiment ... 12

Table S11: Differences in the thickness of cell walls between sun and shade leaves ... 12

Table S12: Differences in the occurrence of necrosis between perforated and unperforated sun leaves. ... 13

Table S13: Differences in the time until necrosis was observed in the different leaf categories. ... 14

Table S14: Differences in the area affected by necrosis in the different leaf categories. ... 14

References ... 14

Mycobiome analyses

An OTU table (Supporting Information Table S1) was generated, coding fungal read counts per sample. Samples with less than 5,000 reads were excluded from statistical analyses. The count data were standardised by total and cumulative sum scaling [1], respectively. Analyses based on the two standardization approaches resulted in comparable results. We therefore present only those based on the standardization by total. To compare mycobiomes between leaf type (sun vs. shade leaves) × feeding damage category we calculated a PERMANOVA based on Bray-Curtis dissimilarity with the Adonis function with 1000 permutations in the R package vegan 2.4-6 [2]. The factors leaf type and feeding damage category were first analysed in crossed designs (single factors and interaction) and then as a combined factor, to disentangle effects caused by the interaction. In both analyses categories are considered to be nested in "tree individual" using the "strata" argument. P-values <0.05 (after Bonferroni correction) were considered to indicate significant differences. The variation explained by each factor was calculated by dividing sum of squares of the factor by the total sum of squares. Subsequently we used the pairwise.adonis function to compare the categories of combined factors and adjusted p-values using Bonferroni correction [3]. We assessed the importance of particular fungal OTUs for the separation of categories by Simper-analyses, which is based on the decomposition of the Bray-Curtis dissimilarity index, with the simper function in vegan package [2].

The number of samples for each sampling category (leaf type × feeding damage category) ranged from 23 to 39 (Supporting Information Table S2), due to limited occurrence of all categories in the field. To compensate for differences in sampling depth and to visualize also minor differences (as indicated by statistical tests) in the abundance of *P. liobae*, the data were transformed as follows: 20 samples were randomly selected of each of the six categories and the proportion of *P. liobae*was noted for each leaf in each run. This was repeated 100 times and each of the resulting 100 subsets of 20 leaves was sorted according to descending proportion of *P. liobae*. The data were plotted as means with 95% confidence levels (box).

Negative controls were applied throughout the study for at least each batch of leaf-mycobiome PCRs. We skipped explicit direct negative controls for the beetle microbiomes, because the results provide indirectly reliable negative controls: Several fungi were detected in the leaf and beetle samples at the same time (i.e. OTU2–7, see supplementary table S1). This confirms that the molecular approach works for both types of samples. We therefore consider the leaf samples as positive controls for detection of OTU1 (i.e. *Petrakia liobae*).

Table S5: Impact of leaf type (sun vs. shade leaves) and feeding damage category on compositional differences of endophytic mycobiomes. Results of PERMANOVA based on Bray-Curtis dissimilarity with the Adonis function with 1000 permutations in the R package vegan 2.4-6 [2]. In both analyses categories are considered to be nested in "tree individual" using the "strata" argument. (A) Leaf type and feeding damage category analysed in crossed designs (single factors and interaction). (B) Leaf type and feeding damage category analysed as a combined factor (only interaction). P-values <0.05 (after Bonferroni correction) were considered to indicate significant differences.

**Factor**	**DF**	**SumsOfSqs**	**MeanSqs**	**F.Model**	**R^2**	**p-value**
**(A)**						
Feeding damage category (FDC)	2	2.868	1.434	3.818	0.042	<0.001
Leaf type (LT)	1	0.394	0.394	1.049	0.006	0.276
FDC × LT	2	0.844	0.422	1.123	0.012	0.249
Residuals	173	64.971	0.376		0.941	
**(B)**						
FDC × LT	5	4.105	0.821	2.186	0.059	<0.001
	173	64.971	0.376		0.941	

Table S6: Pairwise differences in mycobiome composition between leaf categories. Results of a subsequent pairwise adonis analysis (pairwise.adonis function) to compare the categories of combined factors regarding their mycobiomes. Raw and adjusted p-values using Bonferroni correction are shown [3]. Significant (p<0.05, bold) and marginally not significant (p<0.10, bold-italic) differences are highlighted. Leaf type: Shade and Sun leaves; Feeding damage category: No=no damage, Adult=damage by adult *Orchestes fagi* only, Adult/Larvae=damage by adult and larvae of *O. fagi*.

**Pair1**		**Pair2**	**F.Model**	**R^2**	**p-value**	**p-adjusted**
Adult_Shade	vs	No_Shade	1.407	0.023	0.127	1.000
Adult_Shade	vs	Adult_Sun	0.875	0.014	0.545	1.000
Adult_Shade	vs	No_Sun	1.793	0.033	0.030	0.450
Adult_Shade	vs	Adult/Larvae_Sun	1.962	0.030	0.032	0.480
Adult_Shade	vs	Adult/Larvae_Shade	1.964	0.027	0.025	0.375
No_Shade	vs	Adult_Sun	1.177	0.022	0.277	1.000
No_Shade	vs	No_Sun	1.379	0.030	0.129	1.000
**No_Shade**	**vs**	**Adult/Larvae_Sun**	**3.747**	**0.063**	**0.003**	**0.045**
**No_Shade**	**vs**	**Adult/Larvae_Shade**	**3.608**	**0.053**	**0.001**	**0.015**
Adult_Sun	***vs***	***No_Sun***	***2.269***	***0.047***	***0.006***	***0.090***
Adult_Sun	vs	Adult/Larvae_Sun	1.192	0.020	0.275	1.000
Adult_Sun	vs	Adult/Larvae_Shade	1.614	0.024	0.093	1.000
**No_Sun**	**vs**	**Adult/Larvae_Sun**	**4.720**	**0.088**	**0.001**	**0.015**
**No_Sun**	**vs**	**Adult/Larvae_Shade**	**4.847**	**0.078**	**0.001**	**0.015**
Adult/Larvae_Sun	vs	Adult/Larvae_Shade	0.983	0.014	0.436	1.000

Table S7: Contribution of OTUs to compositional differences of mycobiomes in leaves with different feeding damage. Results of a Simper-analysis with the simper function in vegan package [2]to analyse

the importance of particular fungal OTUs for the separation of leaf damage categories; No=no damage, Adult=damage by adult *Orchestes fagi* only, Adult/Larvae=damage by adult and larvae of *O. fagi*. The most important 30 OTUs are shown for each comparison. Average=Average contribution to overall dissimilarity, sd=Standard deviation of contribution, ratio=Average to sd ratio, ava, avb=Average abundances per group, cusum= Ordered cumulative contribution. OTU1 represents *Petrakia liobae*.

	average	sd	ratio	ava	avb	cumsum
**Contrast: Adult_No**						
OTU1	0.126	0.169	0.746	22.690	6.777	0.141
OTU2	0.123	0.139	0.885	14.880	17.890	0.278
OTU3	0.056	0.086	0.649	7.228	6.404	0.340
OTU25	0.053	0.097	0.545	5.108	6.691	0.399
OTU5	0.044	0.107	0.415	4.142	5.377	0.449
OTU10	0.040	0.076	0.529	2.895	7.011	0.493
OTU4	0.038	0.081	0.471	3.549	4.792	0.536
OTU23	0.022	0.094	0.237	1.838	2.738	0.561
OTU9	0.018	0.048	0.378	3.404	0.291	0.581
OTU14	0.015	0.044	0.338	0.910	2.240	0.598
OTU26	0.013	0.065	0.198	0.093	2.492	0.612
OTU16	0.011	0.028	0.381	0.900	1.378	0.624
OTU15	0.010	0.043	0.243	1.935	0.190	0.635
OTU109	0.010	0.067	0.149	0.000	2.003	0.647
OTU12	0.009	0.027	0.343	1.045	0.920	0.657
OTU6	0.009	0.018	0.461	0.967	0.894	0.667
OTU27	0.008	0.039	0.207	0.199	1.443	0.676
OTU98	0.007	0.051	0.147	0.000	1.495	0.684
OTU52	0.007	0.041	0.181	0.240	1.265	0.692
OTU62	0.007	0.049	0.151	0.000	1.488	0.700
OTU11	0.007	0.024	0.313	0.873	0.664	0.709
OTU37	0.007	0.041	0.177	1.110	0.364	0.717
OTU24	0.007	0.031	0.229	0.604	0.841	0.725
OTU35	0.007	0.019	0.354	0.860	0.538	0.732
OTU19	0.006	0.049	0.128	1.252	0.000	0.739
OTU40	0.006	0.048	0.128	1.237	0.000	0.746
OTU128	0.006	0.045	0.128	1.153	0.000	0.752
OTU103	0.006	0.028	0.204	0.268	0.899	0.759
OTU180	0.006	0.034	0.166	0.135	0.988	0.765
OTU17	0.006	0.032	0.176	1.110	0.004	0.771
**Contrast: Adult_Adult/Larvae**						
OTU1	0.189	0.169	1.120	22.690	34.210	0.225
OTU2	0.101	0.138	0.736	14.880	10.160	0.346
OTU3	0.050	0.077	0.659	7.228	5.267	0.406
OTU4	0.040	0.090	0.445	3.549	5.275	0.454
OTU25	0.033	0.093	0.352	5.108	1.826	0.493
OTU5	0.031	0.083	0.374	4.142	2.441	0.530

OTU9	0.027	0.058	0.467	3.404	2.548	0.562
OTU6	0.026	0.057	0.464	0.967	4.708	0.593
OTU10	0.024	0.034	0.714	2.895	3.413	0.622
OTU15	0.021	0.058	0.365	1.935	2.530	0.647
OTU17	0.019	0.053	0.366	1.110	2.959	0.670
OTU11	0.014	0.030	0.464	0.873	2.157	0.687
OTU12	0.013	0.041	0.326	1.045	1.743	0.703
OTU23	0.011	0.063	0.174	1.838	0.370	0.716
OTU22	0.009	0.036	0.253	0.289	1.583	0.727
OTU16	0.009	0.022	0.396	0.900	0.926	0.737
OTU14	0.008	0.020	0.373	0.910	0.706	0.746
OTU24	0.007	0.038	0.192	0.604	0.859	0.754
OTU18	0.007	0.013	0.524	0.395	1.118	0.762
OTU35	0.007	0.017	0.390	0.860	0.558	0.770
OTU19	0.006	0.049	0.128	1.252	0.000	0.778
OTU40	0.006	0.048	0.128	1.237	0.000	0.785
OTU37	0.006	0.041	0.142	1.110	0.051	0.792
OTU128	0.006	0.045	0.128	1.153	0.000	0.799
OTU39	0.005	0.029	0.185	1.023	0.072	0.805
OTU41	0.005	0.021	0.252	0.229	0.876	0.812
OTU46	0.005	0.037	0.143	0.000	1.071	0.818
OTU45	0.005	0.021	0.260	0.828	0.270	0.824
OTU21	0.005	0.042	0.128	1.067	0.001	0.831
OTU28	0.005	0.029	0.172	0.819	0.179	0.837
**Contrast: No_Adult/Larvae**						
OTU1	0.171	0.166	1.030	6.777	34.210	0.192
OTU2	0.109	0.128	0.854	17.890	10.160	0.315
OTU3	0.047	0.069	0.682	6.404	5.267	0.368
OTU4	0.044	0.078	0.562	4.792	5.275	0.417
OTU10	0.040	0.075	0.537	7.011	3.413	0.462
OTU25	0.039	0.073	0.539	6.691	1.826	0.506
OTU5	0.036	0.087	0.415	5.377	2.441	0.547
OTU6	0.026	0.056	0.460	0.894	4.708	0.576
OTU23	0.015	0.075	0.206	2.738	0.370	0.593
OTU17	0.015	0.046	0.324	0.004	2.959	0.610
OTU26	0.015	0.065	0.225	2.492	0.498	0.626
OTU14	0.014	0.044	0.321	2.240	0.706	0.642
OTU9	0.014	0.042	0.327	0.291	2.548	0.658
OTU11	0.013	0.029	0.454	0.664	2.157	0.673
OTU15	0.013	0.043	0.306	0.190	2.530	0.688
OTU12	0.013	0.040	0.318	0.920	1.743	0.702
OTU16	0.011	0.030	0.364	1.378	0.926	0.714
OTU109	0.010	0.067	0.150	2.003	0.003	0.725
OTU27	0.010	0.039	0.254	1.443	0.623	0.737
OTU22	0.008	0.036	0.237	0.136	1.583	0.746
OTU24	0.008	0.045	0.187	0.841	0.859	0.756

OTU62	0.008	0.049	0.158	1.488	0.071	0.764
OTU98	0.007	0.051	0.147	1.495	0.000	0.773
OTU46	0.007	0.039	0.187	0.426	1.071	0.781
OTU52	0.007	0.041	0.180	1.265	0.218	0.789
OTU67	0.007	0.027	0.268	1.105	0.369	0.797
OTU18	0.007	0.013	0.505	0.328	1.118	0.805
OTU35	0.005	0.014	0.367	0.538	0.558	0.811
OTU73	0.005	0.034	0.147	1.003	0.000	0.816
OTU180	0.005	0.033	0.147	0.988	0.000	0.822

Quantitative PCR analyses

Quantification of fungus and host by qPCR was regarded as reliable when 1) the melting curves of both indicated a single PCR product, 2) a PCR product was obtained for *F. sylvatica* and 3) the amplification process was continuous. Samples not fulfilling one of these criteria were discarded. The Cp-value (i.e. the number of the first amplification cycle in which the automatically detected threshold was exceeded) was set to 35 for *P. liobae* if no PCR product was detected until then. Cp-values of the fungus were subtracted from those of beech (ΔCp) to indicate the fungus:host ratio, because PCR is an exponential reaction and higher Cp-values indicate lower DNA-concentrations. To test whether leaf type × feeding damage category affected Cp-value and to test for differences in ΔCp between categories of leaf type × feeding damage we used a linear mixed effects model (lme function in the nlme package) with TreeID as random effect factor. Cp- and ΔCp-values were log-transformed to meet model assumptions. Subsequently we tested for differences among categories using Tukey contrasts (glht function in the multcomp package [4] R2 values were calculated based on Nakagawa et al. [5], and its extension by Johnson [6] in the R package MuMIn [7].

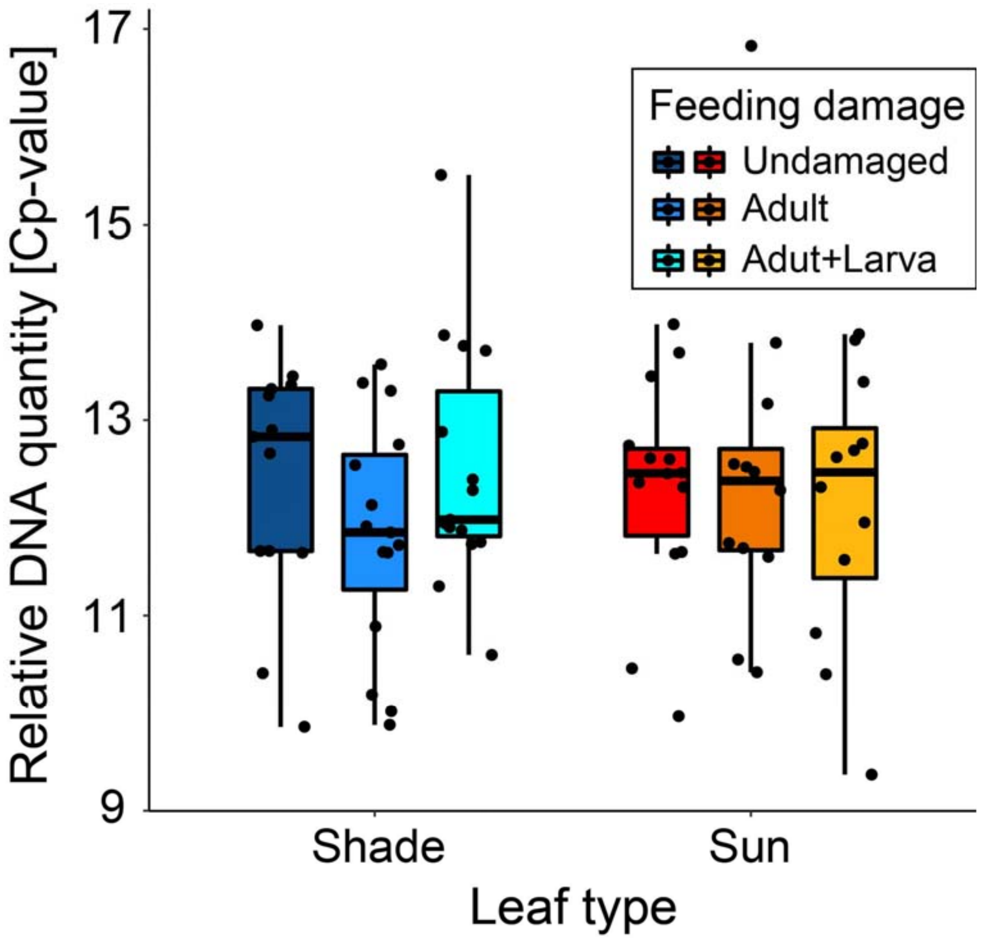

Figure S2: Quantity of beech-DNA across leaf categories. Cp-values obtained with plant-specific primers from the qPCR approach are shown. Median, 25%/75% percentiles (Boxes) and Min-Max values (Whisker) are shown in addition to single data points.

Table S8: Differences in DNA content of the host between leaf categories. Results of a linear mixed effects model (function lme in the nlme package; [8]) testing for differences in CP-beech between leaf type and feeding damage. Tree was used as random effect factor in the model. Results of the anova table are shown. The intercept includes the levels “undamaged” (Feeding damage) and “shade”/”undamaged” (Leaf type × Feeding damage). Marginal R2 (including only fixed effects): 0.0319, conditional R2 (including fixed and random effects): 0.3470.

Variable	numDF	denDF	F-value	p-value
Intercept	1	47	26991.879	<.0001
Leaf type	1	47	0.082	0.7760
Feeding damage	2	47	0.349	0.7073
Leaf type × Feeding damage	2	47	1.377	0.2623

Table S9: Differences in fungus:host ratio between leaf categories. Results of a linear mixed effects model (function lme in the nlme package [8]) testing for differences in pathogen-host ratio (ΔCP) between leaf type × feeding damage categories. Tree was used as random effect factor in the model. ΔCP was log-transformed to meet model assumptions. Results of the anova table are shown. The intercept includes the level “shade”/”adult_damage”. Marginal R2 (including only fixed effects): 0.2615, conditional R2 (including fixed and random effects): 0.3292.

Variable	numDF	denDF	F-value	p-value
Intercept	1	47	10620.354	<.0001
Leaf type × Feeding damage categories	5	47	5.985	<.001

Table S10: Pairwise differences in fungus:host ratio between leaf categories. Post-Hoc comparisons (Multiple Comparisons of Means using Tukey Contrasts) between Leaf type × Feeding damage categories, following the linear mixed effects model described in Table S8 (glht function in the multcomp package [4]).

Pair1		Pair2	Estimate	Std. Error	z-value	adj. p-value
Adult/Larvae_Shade	vs	Adult_Shade	-0.178130	0.078942	2.256	0.21167
No_Shade	vs	Adult_Shade	-0.005257	0.082201	0.064	1.00000
Adult_Sun	vs	Adult_Shade	-0.152850	0.085235	1.793	0.46953
Adult/Larvae_Sun	vs	Adult_Shade	-0.299526	0.084756	3.534	0.00536
No_Sun	vs	Adult_Shade	0.103605	0.080939	1.280	0.79575
No_Shade	vs	Adult/Larvae_Shade	0.172873	0.082491	2.096	0.28880
Adult_Sun	vs	Adult/Larvae_Shade	0.025280	0.084433	0.299	0.99968
Adult/Larvae_Sun	vs	Adult/Larvae_Shade	-0.121396	0.084495	1.437	0.70408
No_Sun	vs	Adult/Larvae_Shade	0.281735	0.080697	3.491	0.00628
Adult_Sun	vs	No_Shade	-0.147593	0.088577	1.666	0.55374
Adult/Larvae_Sun	vs	No_Shade	-0.294269	0.088065	3.341	0.01076
No_Sun	vs	No_Shade	0.108862	0.084288	1.292	0.78949
Adult/Larvae_Sun	vs	Adult_Sun	-0.146676	0.089140	1.645	0.56765
No_Sun	vs	Adult_Sun	0.256455	0.085443	3.001	0.03202
No_Sun	vs	Adult/Larvae_Sun	0.403131	0.085747	4.701	< 0.001

Differences in herbivory between sun and shade leaves

To test for differences in herbivory by adult *O. fagi* between sun and shade leaves we used a linear mixed effects model (lme function in the nlme package [8]) with leaf type as predictor, percentage leaf area loss per leaf as response variable and branch in tree as nested random effect factor (Table S10). Percentage leaf area loss was sqrt-transformed to meet model assumptions. To analyse the probability of attack by adults and larvae separately and combined we used generalised binomial fixed effects models (glmer function in the lmerTest package (Kuznetsova et al 2017)) with number of attacked vs, number of non-attacked leaves (cbind function) as response, leaf type as predictor and branch in tree as nested random effect factor (Table S10).

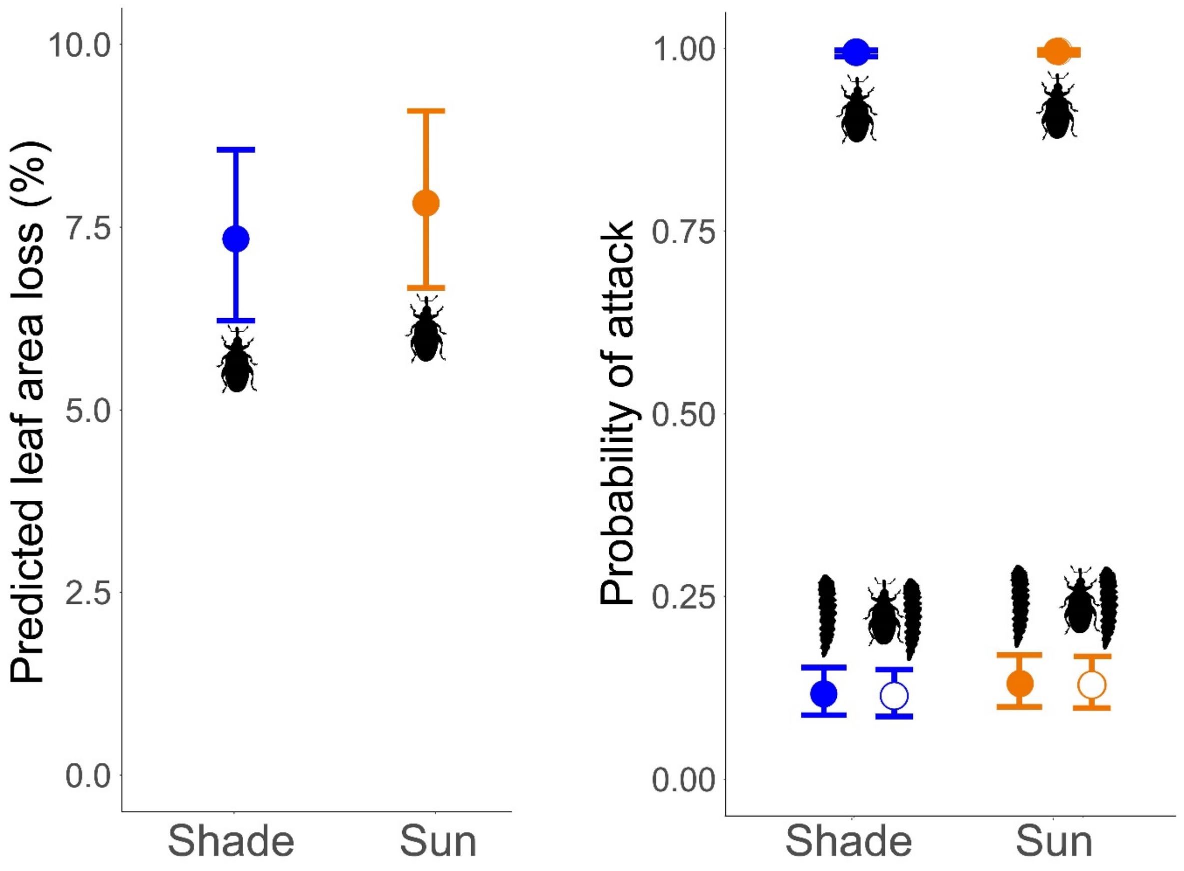

Figure S1: Probabilities (means, CI) of the extent of feeding damage by adults of *Orchestes fagi* (left) as well as probability of attack by adults, larvae and combined attack of adult/larvae of *Orchestes fagi* (right) on sun (N=2747) and shade (N=2146) leaves. Probabilities are based on either a linear mixed effects model (response: sqrt (proportion leaf area loss; lme function in the nlme package [8]; left) or binomial generalized mixed effects models (response: attacked leaves vs. non-attacked leaves, function glmer in the lmerTest package REF; right) using the emmeans function in the "lsmeans" package. The nested structure (branch in tree) was considered in the random term of the models.

Table S2: Differences in herbivory by *Orchestes fagi* between sun and shade leaves. Results of (A) a linear mixed effects model (function lme in the nlme package (Pinheiro et al 2016)) testing for differences in herbivory by *O. fagi* between sun and shade leaves, and (B)-(D) generalised binomial

fixed effects models (function glmer in the lmerTest package (Kuznetsova et al 2017)) testing for probabilities of attack . Branch in Tree was used as a nested random effect factor in all models. Results of the anova tables are shown.

(A) Percentage leaf area loss (sqrt transformed)

**Variable**	**numDF**	**denDF**	**F-value**	**p-value**	**Effect direction**
Intercept	1	5436	688.5313	<.0001	
Leaf type	1	5436	14.2780	<.001	Sun>Shade

Marginal R2 (including only fixed effects): 0.002
Conditional R2 (including fixed and random effects): 0.482

(B) Probability of attack (adults)

Variable	numDF	denDF	z-value	p-value	Effect direction
Intercept	1	103	13.593	<.0001	
Leaf type	1	103	1.123	0.261	

Marginal R2 (including only fixed effects): 0.008
Conditional R2 (including fixed and random effects): 0.974

(C) Probability of attack (larvae)

Variable	numDF	denDF	z-value	p-value	Effect direction
Intercept	1	103	-12.745	<.0001	
Leaf type	1	103	1.616	0.106	

Marginal R2 (including only fixed effects): 0.005
Conditional R2 (including fixed and random effects): 0.924

(D) Probability of attack (adults & larvae)

Variable	numDF	denDF	z-value	p-value	Effect direction
Intercept	1	103	-12.847	<.0001	
Leaf type	1	103	1.714	0.087	Sun>Shade

Marginal R2 (including only fixed effects): 0.006
Conditional R2 (including fixed and random effects): 0.923

Incubation Experiment

In general, plants use two defense strategies against herbivores, damaged self-recognition and herbivore associated molecular patterns (HAMPs) [9]. First is seen as a more ancient mechanism and provides a more general response, and second as more derived mechanism that allows a more rapid and intensive response, in particular against specialist enemies [10]. We assumed HAMPs to be more important in the responses to the specialist herbivore *O. fagi*. By punching detached leaves, we aimed to exclude animal-derived elicitors as well as systemic responses of beech and regulation of tree and stomata processes.

For experimental infection, one branch in each of the sun-exposed and shaded canopy of 10 mature beech trees, was sampled on 29 August 2017. Two thirds of the 15 visually healthy and undamaged leaves chosen of each tree and leaf type (sun vs. shade leaves) were perforated using a sterile iron hollow punch (3 mm in diameter). It was executed at the four corners of a virtual rectangle, with edge lengths of roughly half the leaf length and width, respectively. For inoculation, we cut small agar cylinders (height 5mm, ø 2.5 mm) with young active mycelium of *P. liobae* from the growing zone of the cultures using sterile plastic tubes (drinking straws). Two cylinders were placed on each leaf. For perforated leaves, the mycelia were positioned in immediate proximity of two of the four holes. The leaves were incubated in sterile, transparent plastic boxes (15 cm × 13.5 cm × 6 cm) on moistened paper tissue generating 100 % air humidity in a climate camber at 20 °C under permanent light. Each box contained one leaf per treatment of the same tree and leaf type (total: 2 leaf types × 10 trees × 5 replicates = 100 boxes). Leaves were digital-photographically documented twice per week and occurrence of necroses and mycopappi were noted. After 11 days of incubation, when necrotic areas were quantified, most leaves showed necroses but no indications of senescence, except for twelve leaves which were excluded from further analyses. After 30 days, the experiment was terminated because moulds grew on most leaves.

We tested for differences in the thickness of the outer cell walls of upper epidermis cells in shade and sun leaves using a linear mixed effects model (lme function in the nlme package [8]) with cell wall thickness as response, leaf type as predictor and tree as random effect factor (Table S11).

Table S11: Differences in the thickness of cell walls between sun and shade leaves. Results of a linear mixed effects model (function lme in the nlme package (Pinheiro et al 2016)) testing for differences in cell wall thickness between sun and shade leaves. Tree was used as a random effect factor in the model. Cell wall thickness was log-transformed to meet model assumptions. Results of the anova table are shown. Marginal R2 (including only fixed effects): 0.824, conditional R2 (including fixed and random effects): 0.882.

Variable	numDF	denDF	F-value	p-value	Effect direction
Intercept	1	69	3918.551	<.0001	
Leaf type	1	69	550.307	<.0001	Sun>Shade

As measure of *P. liobae* infection we used three different parameters: (1) proportion of leaves infected, (2) time until necrosis appears, and (3) size of the necrosis after 11 days. As all shade leaves were infected in our experiment, a model testing for effects of treatment (perforated vs. unperforated) and leaf type (sun vs. shade leaves) and their interaction on the infection of leaves was not possible. We tested only the effect of perforation in sun leaves. We used a binomial generalized

linear mixed effects model (function glmer in the lmerTest package [11]) with infection (0/1) as response, treatment (perforated vs. unperforated) as predictor, and leaf in tree as nested random effect factor (Table S12). Only infected leaves were used to test for differences in times until necrosis appears and the size of the necrosis with a linear mixed effects model (lme function in the nlme package); defining time until necrosis or size of the necrosis as response, leaf type (sun vs. shade leaves) and treatment (perforated vs. unperforated) and their interaction as predictor and leaf (two infection trials per leaf) in tree as nested random effect factor (Tables S13–14).

Table S12: Differences in the occurrence of necrosis between perforated and unperforated sun leaves. Results of a binomial generalized linear mixed effects model (function glmer in the lmerTest package [11]) testing for differences in the infection between perforated and unperforated sun leaves (occurrence of necrosis). Leave in tree was used as a nested random effect factor in the model. Results of the anova table are shown. Marginal R2 (including only fixed effects): 0.073, conditional R2 (including fixed and random effects): 0.982.

**Variable**	**numDF**	**denDF**	**Estimate**	**Std. Error**	**z-value**	**p-value**	**Effect direction**
Intercept	1	198	15.745	2.760	5.70	<.0001	
Treatment	1	198	7.305	1.483	4.927	<.0001	perforated>unperforated

Table S13: Differences in the time until necrosis was observed in the different leaf categories. Results of a linear mixed effects model (function lme in the nlme package; [8]) testing for effects of leaf type (sun, shade leaves), treatment (perforated, unperforated) and their interaction on the time until necrosis was observed. Leave in tree was used as a nested random effect factor in the model. Results of the anova table are shown. The intercept includes the levels “shade”/”perforated” (Leaf type × Treatment). Marginal R2 (including only fixed effects): 0.530, conditional R2 (including fixed and random effects): 0.612.

Variable	numDF	denDF	F-value	p-value	Effect direction
Intercept	1	317	1315.9071	<.0001	
Leaf type	1	317	495.3671	<.0001	Sun>Shade
Treatment	1	317	0.2243	0.6361	
Leaf type × Treatment	1	317	1.4263	0.2333	

Table S14: Differences in the area affected by necrosis in the different leaf categories. Results of a linear mixed effects model (function lme in the nlme package; [8]) testing for effects of leaf type (sun, shade leaves), treatment (perforated, unperforated) and their interaction on the area affected by necrosis. Leave in tree was used as a nested random effect factor in the model. Results of the anova table are shown. The intercept includes the levels “shade”/”perforated” (Leaf type × Treatment). Marginal R2 (including only fixed effects): 0.414, conditional R2 (including fixed and random effects): 0.579.

Variable	numDF	denDF	F-value	p-value	Effect direction
Intercept	1	335	362.6806	<.0001	
Leaf type	1	335	359.1655	<.0001	Sun<Shade
Treatment	1	335	11.5255	<.00081	perforated>unperforated
Leaf type × Treatment	1	335	6.4738	0.0114	

References

1. Paulson JN, Stine OC, Bravo HC, Pop M. Differential abundance analysis for microbial marker-gene surveys. Nat Methods. 2013;10:1200–2. doi:10.1038/nmeth.2658.
2. Oksanen J, Blanchet FG, Friendly M M, Kindt R, Legendre P, McGlinn D, et al. vegan: Community Ecology Package: R package version 2.4-6.; 2018.
3. Martinez Arbizu P. pairwiseAdonis: Pairwise multilevel comparison using adonis.: R package version 0.0.1.; 2017.
4. Hothorn T, Bretz F, Westfall P. Simultaneous inference in general parametric models. Biom J. 2008;50:346–63. doi:10.1002/bimj.200810425.
5. Nakagawa S, Schielzeth H, O’Hara RB. A general and simple method for obtaining R2 from generalized linear mixed-effects models. Methods Ecol Evol. 2013;4:133–42. doi:10.1111/j.2041-210x.2012.00261.x.
6. Johnson PC. Extension of Nakagawa & Schielzeth’s R2GLMM to random slopes models. Methods Ecol Evol. 2014;5:944–6. doi:10.1111/2041-210X.12225.

7. Barton K. MuMIn: Multi-Model Inference: R package. https://CRAN.R-project.org/package=MuMIn; 2019.
8. Pinheiro J, Bates D, DebRoy S, Sarkar D, R Core Team. nlme: Linear and Nonlinear Mixed Effects Models: R package version 3.1-128; 2016.
9. Wu S, Zhu Z, Fu L, Niu B, Li W. WebMGA: A customizable web server for fast metagenomic sequence analysis. BMC Genomics. 2011;12:444. doi:10.1186/1471-2164-12-444.
10. Heil M, Ibarra-Laclette E, Adame-Álvarez RM, Martínez O, Ramirez-Chávez E, Molina-Torres J, Herrera-Estrella L. How plants sense wounds: damaged-self recognition is based on plant-derived elicitors and induces octadecanoid signaling. PLoS ONE. 2012;7:e30537. doi:10.1371/journal.pone.0030537.
11. Kuznetsova A, Brockhoff PB, Christensen RHB. lmerTest Package: Tests in Linear Mixed Effects Models. J. Stat. Soft. 2017. doi:10.18637/jss.v082.i13.
